# Elucidating the Therapeutic Potential of Bis(Maltolato)OxoVanadium(IV): The Protective Role of Copper in Cellular Metabolism

**DOI:** 10.3390/ijms24119367

**Published:** 2023-05-27

**Authors:** Lorenzo Rivas-García, Alfonso López-Varela, José L. Quiles, María Montes-Bayón, Pilar Aranda, Juan Llopis, Cristina Sánchez-González

**Affiliations:** 1 Department of Physiology, Institute of Nutrition and Food Technology “José Mataix Verdú”, Biomedical Research Centre, University of Granada, 18016 Armilla, Spain; lorenrivas@ugr.es (L.R.-G.); alvarela@ugr.es (A.L.-V.); jlquiles@ugr.es (J.L.Q.); paranda@ugr.es (P.A.); crissg@ugr.es (C.S.-G.); 2 Sport and Health Research Centre, University of Granada, C/Menéndez Pelayo 32, 18016 Granada, Spain; 3 Research and Development Functional Food Centre (CIDAF), Health Science Technological Park, Avenida del Conocimiento 37, 18016 Granada, Spain; 4 Research Group on Foods, Nutritional Biochemistry and Health, Universidad Europea del Atlántico, Isabel Torres 21, 39011 Santander, Spain; 5 Instituto de Investigación Sanitaria del Principado de Asturias (ISPA), Department of Physical and Analytical Chemistry, Faculty of Chemistry, University of Oviedo, C/ Julián Clavería 8, 33006 Oviedo, Spain; montesmaria@uniovi.es

**Keywords:** vanadium, copper, mineral, nutrition, mitochondria, DNA

## Abstract

Vanadium (V) is a trace mineral whose biological activity, role as a micronutrient, and pharmacotherapeutic applications remain unknown. Over the last years, interest in V has increased due to its potential use as an antidiabetic agent mediated by its ability to improve glycemic metabolism. However, some toxicological aspects limit its potential therapeutic application. The present study aims to evaluate the effect of the co-treatment with copper (Cu) and bis(maltolato)oxovanadium(IV) (BMOV) as a possible strategy to reduce the toxicity of BMOV. Treating hepatic cells with BMOV reduced cell viability under the present conditions, but cell viability was corrected when cells were co-incubated with BMOV and Cu. Additionally, the effect of these two minerals on nuclear and mitochondrial DNA was evaluated. Co-treatment with both metals reduced the nuclear damage caused by BMOV. Moreover, treatment with these two metals simultaneously tended to reduce the ND1/ND4 deletion of the mitochondrial DNA produced with the treatment using BMOV alone. In conclusion, these results showed that combining Cu and V could effectively reduce the toxicity associated with V and enhance its potential therapeutic applications.

## 1. Introduction

Vanadium (V) is a widely distributed element in nature, and it is essential for some life forms. However, several aspects of V metabolism, such as its absorption, distribution, and biological and pharmacological activities still need to be fully defined. Currently, the research on V has increased, motivated by its increasing environmental levels due to its wide use in industrial processes [1], its use as a supplement for athletes, and the growing interest in the pharmacological applications of some vanadium compounds as hypoglycemic agents. However, before its use in the clinic, many metabolic aspects must be well established, such as digestive and metabolic interactions with other elements involved in the antioxidant defense [2].

During the last few years, multiple chemical compounds of V have been evaluated. Finally, bis(maltolato)oxovanadium(IV) (BMOV) is the V compound that reports the highest activity (i.e., BMOV is 2 to 3 times more potent than inorganic V as a glucose-lowering agent than vanadium sulfate), and the chemical V compound reporting the best tolerance [3]. In addition, BMOV can be administered to experimental animals in drinking water with similar effectiveness as vanadium sulfate [4]. BMOV has shown the ability to decrease fasting glucose and hemoglobin A1C levels in diabetic rats [5]. Moreover, treatment with V decreased the exogenous insulin necessity of diabetic rats, improving insulin sensitivity [6]. The antidiabetic mechanism associated with V could be mediated mainly by two effects. On the one hand, the vanadyl ion could stimulate cytosolic protein kinases and subsequently improve cellular signaling. On the other hand, another mechanism proposed is mediated by the translocation of the GLUT-4 glucose transporter from the intracellular compartment to the plasma membrane [7]. However, several limitations reduce the use of V in the clinic as an antidiabetic agent. The main limitation is the ability of V to generate reactive oxygen species (ROS) during metabolism [8]. ROS are molecules that can cause cellular damage and inflammation, leading to other complications. V stimulates the production of ROS in certain tissues [9], e.g., exposure to vanadium pentoxide (V^5+^) is correlated with an oxidative biomarker, and this exposure causes fibroblast senescence and pulmonary fibrosis [10]. Moreover, the exposure of hepatic cells to BMOV causes damage to cellular DNA and mitochondrial metabolism [11].

Thus, several strategies to reduce V toxicity are required. In that way, combining V with other metallic elements has emerged as an innovative solution. Magnesium could reduce the prooxidant activity of V [12], and the combination of BMVO with MnCl_2_ has revealed that this agent can decrease the damage produced by V in the nucleus and mitochondria [11].

Copper (Cu) is a transition metal whose main function in biological systems is the regulation of enzymatic processes [13]. The role of this mineral in antioxidant defense is noteworthy because it is a cofactor of several enzymes involved in this process [14]. Cu is involved in the enzymes superoxide dismutase (SOD) located in the cytosol [15], lysyl oxidase [16] in the connective tissue, and cytochrome C oxidase [16], which catalyzes the reaction between oxygen and hydrogen peroxide during cellular respiration. Cu exerts its antioxidant action mainly due to its role in Cu/Zn SOD [15], a metalloenzyme that protects against oxidative damage. Cu/Zn-SOD is located in the cytosol of most tissues and is an integral part of an organism’s defense mechanism against the consequences of oxygen metabolism.

The interactions between Cu and V have yet to be elucidated. Several authors found, in general, no significant differences between Cu absorption and V compound absorption [17]. However, other authors suggest that V treatment may affect Cu metabolism in different tissues. For instance, V treatment significantly decreased Cu in tissues such as the liver and kidney [12]. Nevertheless, treatment with V increased the amount of Cu in the femur [18]. This association could be because the absorption of these metals occurs through a common process mediated by divalent metal transporter (DMT1) [19,20].

The present study aims to evaluate the effect of adding Cu on cell viability and the mitochondrial and nucleus DNA stability of cells previously treated with BMOV. Thus, these findings will provide new strategies for implementing V in therapy.

## 2. Results

### 2.1. Cell Viability (MTT Assay)

**Figure 1:**
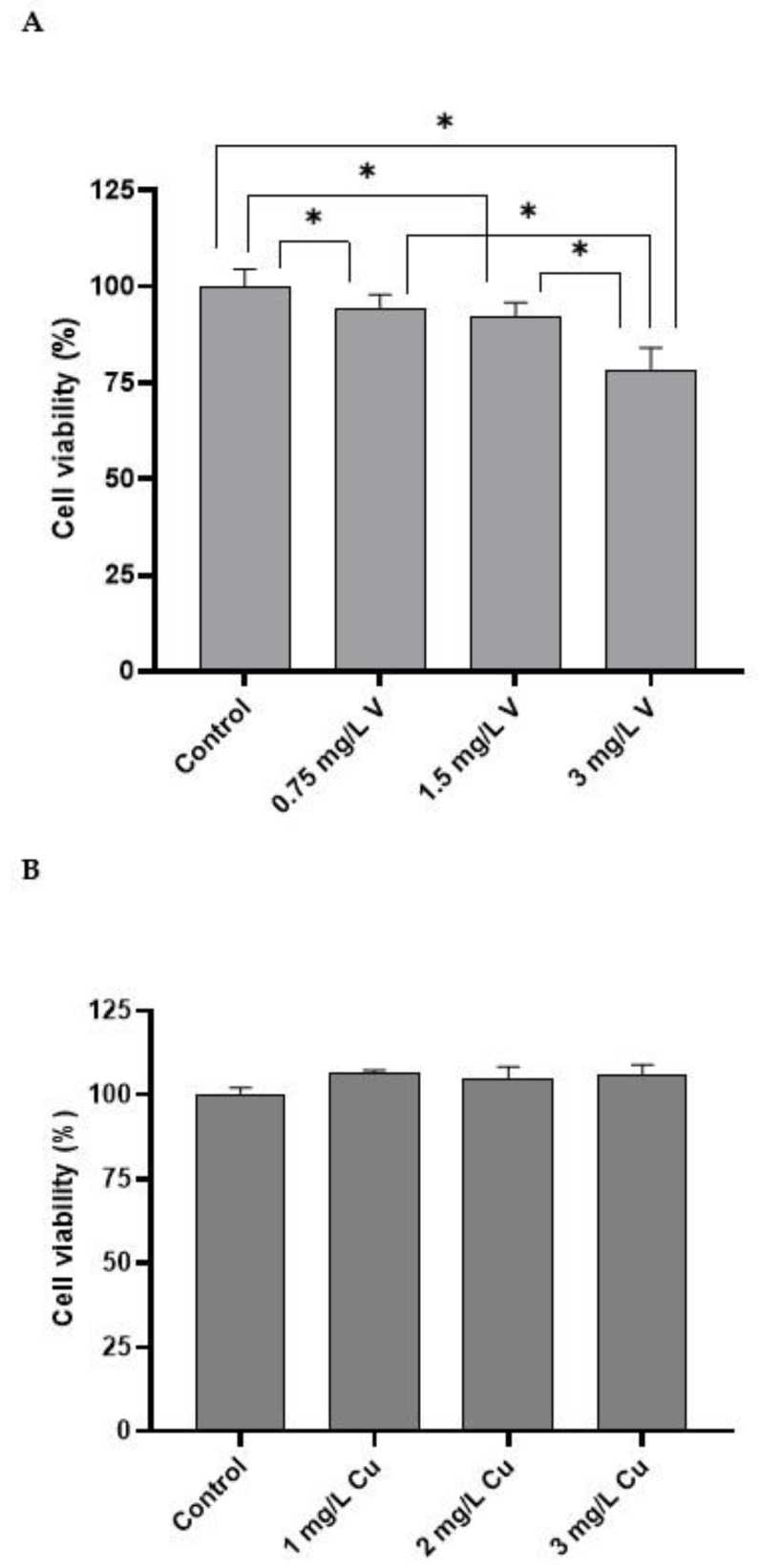

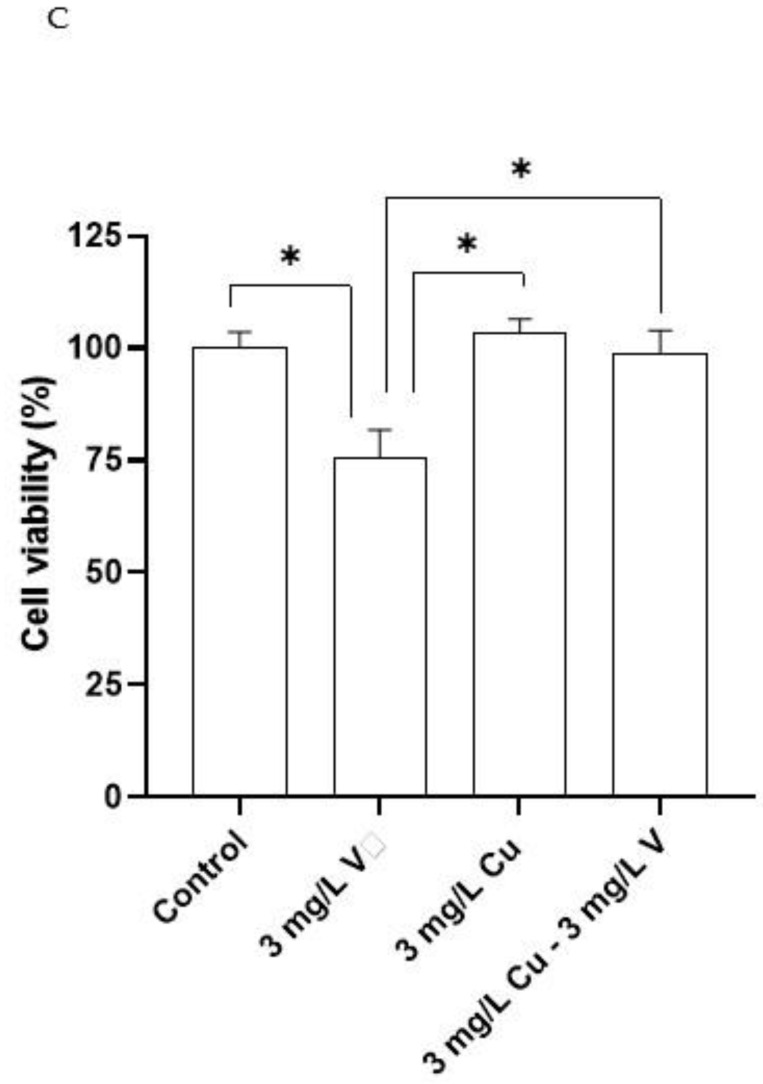
Cell viability of HepG2 cells. (**A**): Cells treated with different doses of BMOV; (**B**): cells treated with different doses of CuCl_2_; (**C**): cells treated with the combination of both metals. * *p* < 0.05. ns.: nonsignificant. Results are presented as the mean ± SD.

Figure 1A shows the effect of V treatment (as BMOV) on hepatic cells for 32 h. The intermediate doses (0.75 mg/L V and 1.5 mg/L V) significantly decreased cell viability compared with the control cells. The highest dose (3 mg/L V) significantly decreased cell viability compared with all the other groups.

However, no changes were found in cell viability after treating with CuCl_2_ for 32 h for all the doses studied compared with the control cells (Figure 1B). Moreover, combining both metals (Figure 1C) showed no modification in cell viability compared with the control cells.

### 2.2. Metal Uptake

The amounts of V and Cu were measured using the inductively coupled plasma mass spectrometry technique (ICP-MS). Treatment with 3 mg/L V (as BMOV) increased the cellular content of V and Cu compared with control cells (Table 1). Moreover, if cells were exposed to 3 mg/L Cu (as CuCl_2_), it increased the uptake of Cu (Table 1). Furthermore, when cells were exposed to the combination of metals at the same time, the uptake of Cu increased, but less than after the exposure to Cu alone, and the uptake of V was similar to that found after treatment with BMOV alone.

### 2.3. ND1/ND4 mtDNA Deletion

**Figure 2:**
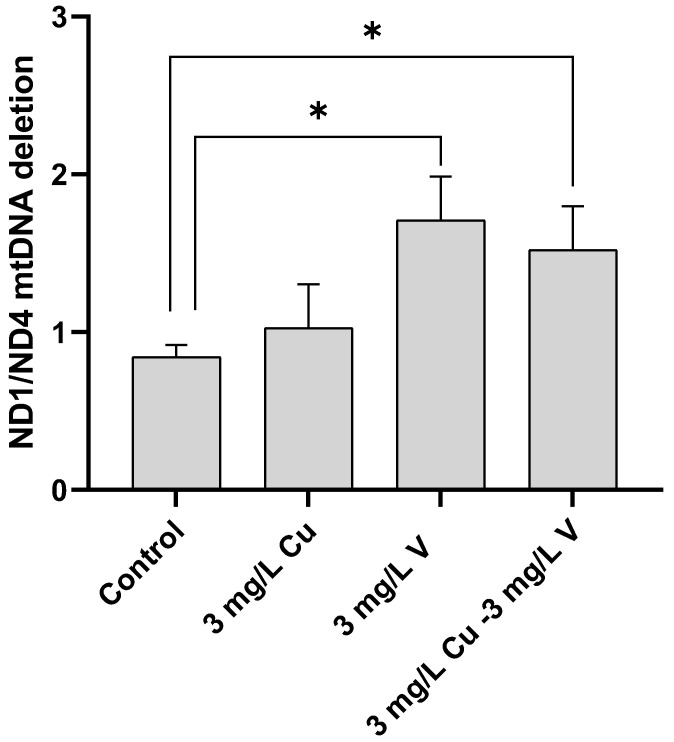
ND1/ND4 mtDNA deletion in HepG2 cells treated with metallic solutions for 32 h. * *p* < 0.05.

ND1/ND4 deletion was measured to evaluate the mtDNA status. Control cells and cells treated with 3 mg/L Cu for 32 h showed no significant differences in this parameter. In contrast, cells treated with 3 mg/L V and the combination (3 mg/L Cu–3 mg/L V) showed a significant increase in ND1/ND4 mtDNA deletion compared to control cells and cells treated with 3 mg/L Cu (Figure 2). No significant difference was found between cells treated with 3 mg/L V and cells treated with the combination (3 mg/L Cu–3 mg/L V).

At our experimental conditions, the combined treatment of 3 mg/L Cu–3 mg/L V showed a tendency to reduce ND1/ND4 mtDNA deletion compared to the treatment with BMOV alone (3 mg/L V).

### 2.4. Comet Assay

**Figure 3:**
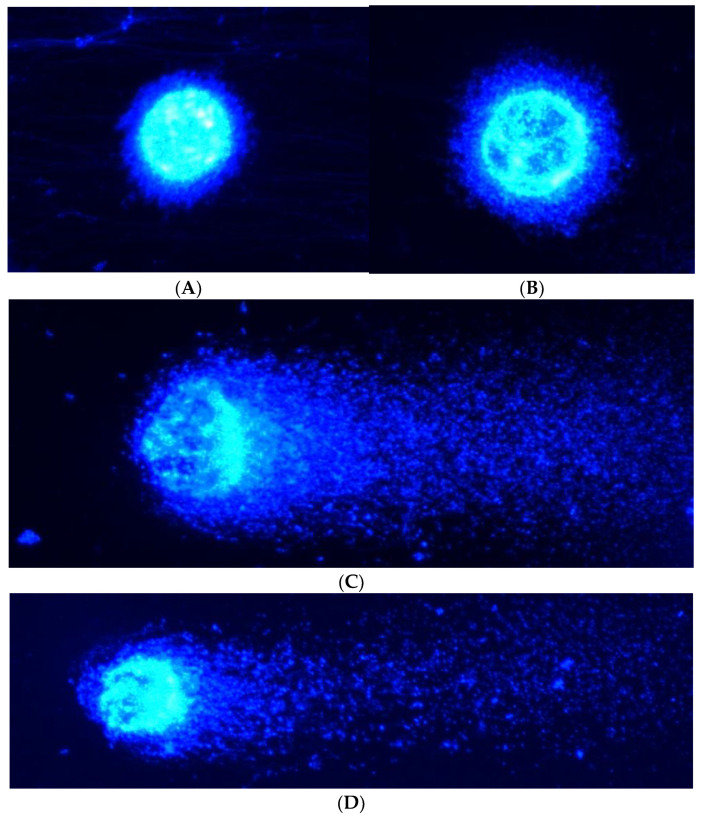
Comets from HepG2 cells (20x). (**A**) Control cells; (**B**) cells treated with 3 mg/L Cu; (**C**) cells treated with 3 mg/L V; and (**D**) cells treated with 3 mg/L Cu–3 mg/L V.

The comet assay was conducted to evaluate the nuclear DNA damage (Table 2). The Olivé tail moment (OTM) was the parameter used to compare group differences. Table 2 shows no differences between the cells treated with 3 mg/L Cu and control cells. In contrast, those treated with 3 mg/L V had an OTM value almost 40 times higher than the control cells and the cells treated with 3 mg/L Cu. The cells treated with 3 mg/L V exhibited the highest OTM score. Additionally, the combination of metals (3 mg/L Cu–3 mg/L V) produced an OTM value of 10.3, which was statistically lower than the group treated with 3 mg/L V and statistically higher than both the control group and the group treated with 3 mg/L Cu. Figure 3 shows a representative image for each treatment from the comet assay.

## 3. Discussion

Firstly, the effect of the addition of the metals studied was assessed using the MTT test, which is widely used to evaluate the impact of chemical compounds and drugs on cell metabolism. The forms of each metal used were the chemical compound with the highest therapeutic potential; BMOV was used for testing the V addition, and CuCl_2_ was used for Cu. Moreover, a model of hepatic cells (HepG2) was used because the liver is the main organ responsible for metabolism in mammals. This cellular model has been previously used in other studies to evaluate the uptake, biological effect, and toxicity of metallic elements [11,21,22]. However, this cell line exhibits limitations compared with primary hepatocytes, such as low metabolic capacities, i.e., lower expression of some metabolic activities [23].

Under our experimental conditions, the incubation of hepatic cells with different doses of BMOV resulted in a significant decrease in cell viability compared with the control cells (Figure 1A). The significant decrease associated with the highest dose (3 mg/L V) is particularly relevant. These doses of V are similar to those obtained in the plasma of hyperglycemic rats treated with BMOV for 5 weeks [24] and are effective doses for lowering blood glucose levels. In fact, under these conditions, treatment with BMOV reduced hepcidin levels [25], increased intracellular erythropoietin signaling [25], and restored non-iron deficiency anemia [25]. However, the present study reported a toxic effect associated with the incubation with BMOV, which may be related to the prooxidant and pro-inflammatory effects reported in experimental animals [24,26,27]. It could also be related to the hepatotoxicity reported by other authors in experimental animals [28]. Furthermore, the effect of Cu addition was tested, and in this case, an increase in cell viability was observed for all studied doses (Figure 1B). This effect can be associated with the antioxidant capacity of Cu. Thus, the form of SOD located in the cytoplasm and nucleus requires the presence of Cu for its catalytic center to be active [15]. Therefore, an extra supply of Cu will promote the action of SOD and protect cells from potential free radicals, increasing their viability [15].

Subsequently, the effect of both metals on cellular metabolism was evaluated, and in this case, the toxic effect of BMOV was reduced by Cu (Figure 1C). The effect of decreasing BMOV toxicity thanks to supplementation with antioxidant metals has been previously reported. Rivas-García L et al. described that Mn addition (as MnCl_2_) decreased BMOV toxicity by acting through the mitochondria. It has also been observed that Cu can protect other tissues from toxic effects mediated by other metals, such as aluminum [29].

The cellular uptake activity was monitored to further understand the cellular metabolism of both metals. Both metals could be transported through the cell membrane by the same transporter, DMT1 [19,20]. However, the information available on the capacity of V to modify the expression of the transporter is limited. Similarly, it is unknown whether overexposure to V could alter the functionality of the transporter [19]. Ścibior et al. described that the expression of DMT1 was not modified with V supplementation in the liver of rats, while in other tissues, such as the kidney, it was decreased [12]. Under the present conditions, individual exposure to hepatocytes increased the content of both metals (Table 1). Additionally, BMOV exposure increases the individual content of Cu. Thus, the presence of BMOV mediates the entry of Cu into cells. This same effect has been previously described because BMOV exposure increased the uptake of another antioxidant metal (Mn) [11]. These results are consistent with others in which only increases in Cu have been found in the kidneys of rats treated with V [30]. The co-exposure of hepatocytes to both metals led to repetition in the trends for individual treatments. Thus, when cells were exposed to BMOV and Cu in combination, the intracellular levels of both elements increased without reaching the values observed in individual exposures. In this case, the effect of Cu and BMOV administration was contrary to Mn and BMOV supplementation; in that case, the presence of Mn in the medium facilitated the exit of the other mineral elements [11]. However, in other in vivo studies, treatment with sodium metavanadate decreased the content of Cu in the liver, kidney, and spleen [12]. Furthermore, treatment with sodium metavanadate has also been described to potentially increase the content of Cu in a femur diaphysis [18].

Subsequently, ND1/ND4 deletion of mitochondrial DNA (mtDNA) was studied. ND1 and ND4 are subunits of mtDNA that encode complex I [31], one of the largest and most complicated protein complexes found in mitochondria. Mutations in these genes can lead to mitochondrial diseases, and studies have shown that certain mutations in ND1 and ND4 can cause complex I dysfunction, which can contribute to various health conditions [32]. Therefore, understanding the relationship between ND1/ND4 and complex I is crucial for understanding the complex biology of mitochondria and its role in cellular energy production [33].

The data reported are consistent with previously published findings [11]. Under the present conditions, the treatment with BMOV increased ND1/ND4 deletion in mtDNA (Figure 2), which could be associated with mitochondrial dysfunction, probably caused by the prooxidant damage induced by BMOV. However, treatment with Cu showed no influence on the ND1/ND4 deletion compared with control cells. These results are partially inconsistent with recent publications, which suggest that Cu plays a crucial role in mitochondrial function and signaling, affecting cell fate through metabolic reprogramming involving bioenergetics, dynamics, and mitophagy [14,34].

When the combined treatment was applied, the deletion of ND1/ND4 tended to increase compared with control cells, but to a lesser extent than with V treatment, indicating a corrective effect produced by Cu (Figure 2).

Finally, the impact of these metals on nuclear DNA was evaluated. Thus, the ROS generated by BMOV treatment interacted with nuclear DNA. In addition, the treatment with CuCl_2_ showed no effect on the nuclear structure. When both metals were added together, the nuclear damage was reduced compared with BMOV treatment alone; this could be associated with the antioxidant activity of Cu.

## 4. Materials and Methods

### 4.1. Preparation of Metallic Solutions and Exposure to V and Cu

BMOV was used as the complex of V and CuCl_2_ as the source of Cu. The preparation of the BMOV complex was extemporaneous. CuCl_2_ (Sigma Aldrich, St. Louis, MO, USA) was prepared in water to the concentration required. BMOV was synthesized according to the protocol described by Peters, K. et al. [35]. Briefly, the complex was synthesized by adding vanadyl sulfate and ligand to water and adjusting the pH to 9. The stability of the BMOV in the cell culture medium was previously determined [11].

Cells were incubated with BMOV at the following concentrations of V: 0.75, 1.5, and 3 mg/L V. For Cu exposure, cells were incubated with CuCl_2_ at the following concentrations of Cu: 1, 2, and 3 mg/L Cu. The selection of doses in the present study was calculated based on the serum concentration found in vivo [24,27]. The Cu concentrations correspond to the physiological levels of Cu in the plasma of rats, twofold and threefold. MTT tests were performed at all the proposed concentrations of metals. However, only the highest concentration of each metal and the combination of both metals were used for the metallic uptake, mtDNA deletion, and comet assay. Cells were incubated with the metals for 32 h in all the experiments.

### 4.2. Cell Conditions

The HepG2 cell line was supplied by the Cell Culture Resource Centre at the University of Granada (Spain). Cells were precultured in 25 cm^2^ culture flasks (Thermo Fisher Scientific, Waltham, MA, USA) at 37 °C using RPMI 1640 medium (Sigma Aldrich, St. Louis, MO, USA) supplemented with 10% (*v*/*v*) fetal bovine serum and 2 mM glutamine in a humidified atmosphere of 5% CO_2_. The culture medium for HepG2 was replaced once per day. After the cell density reached approximately 1 × 10^6^ cell/mL, cells were detached with trypsinization using a 0.25% trypsin-EDTA solution (Sigma Aldrich, St. Louis, MO, USA) and collected using centrifugation at 1500 rpm for 5 min. Cell density was determined using a Neubauer chamber.

### 4.3. MTT Assay

Cell viability was evaluated by assessing the production of formazan by viable cells from a salt (3-(4,5-dimethylthiazol-2-yl)-2,5-diphenyltetrazolium bromide) (MTT). Briefly, cells were seeded at 1 × 10^5^ cells per mL in 96-well plates (VWR, Radnor, PA, USA) and incubated for 24 h. Then, the media were refreshed, and the appropriate dilutions of each metallic solution were added. After incubation for 32 h, the medium was discarded and replaced with a 0.5 µg/mL solution of MTT (Sigma, St. Louis, MO, USA) prepared in PBS (Phosphate Buffered Saline, Sigma, St. Louis, MO, USA) for 1 h at 37 °C. Then, the MTT was removed, and DMSO was added to solubilize the formed crystals. Last, absorbance values were measured at 555 nm (555–690) using a microplate reader (Agilent Technologies, Winooski, VT, USA). All experiments were performed in triplicate.

### 4.4. Metal Uptake

HepG2 cells were seeded at 1 × 10^5^ cells/mL in a 25 cm^2^ cell culture flask. After 24 h, the medium was replaced, and cells were treated with the metallic solutions for 32 h. Then, hepatocytes were washed with PBS and diluted with a basic solution containing ammonium hydroxide, butanol, EDTA, and Triton X-100. The quantification of V and Cu was performed using an ICP-MS instrument (Agilent 7500, Agilent Technologies, Winooski, VT, USA) fitted with a Meinhard type nebulizer (Glass Expansion, Weilburg, Germany) and equipped with a He collision cell. A Milli-Q system (Millipore, Burlington, MA, USA) was used to obtain deionized water (18 MΩ). All reagents (ammonium hydroxide solution, butanol, EDTA, Triton X-100) used were of the highest available purity. A standard solution of 100 μg/L of Li, Mg, Sc, Co, Y, In, Ce, Ba, Pb, Bi, and U in 1% (*v*/*v*) HNO3 was prepared from a 1000 mg/L multi-element stock standard solution (Merck, Darmstadt, Germany) and used for daily optimization of the ICP-MS parameters. Single-element standard solutions for ICP-MS containing 1000 μg/mL of V and Cu were also provided by Merck (Merck, Darmstadt, Germany). Calibration curves were prepared using Ga as an internal standard and by diluting stock solutions of 1000 mg/L in 1% HNO_3_. The accuracy of this method was evaluated by comparing it with the certified reference material SeronormTM Trace Elements Serum (Sero, Billingstad, Norway) and using recovery studies of spiked samples with multi-element standards. The calculated recoveries for each element were between 95% and 105% in all cases. For each element, a mean of five separate determinations of this reference material was used.

### 4.5. ND1/ND4 mtDNA Deletion

HepG2 cells were exposed to the metallic solutions as described in Section 2.4. Then, ND1/ND4 deletion was measured using the previously proposed methodology [36].

### 4.6. Comet Aassay

Hepatocytes were allowed to grow until 60% confluence was reached. Subsequently, cell cultures were exposed to the metallic solutions for 32 h. Then, cells were treated with trypsin–EDTA solution (Sigma, St Louis, MO, USA) and centrifuged for pellet collection. Cells were resuspended in 1 mL PBS. Microscope glass slides were pre-coated with 1% normal melting point (NMP) agarose on one side. A total of 30 μL of cell suspension were mixed with 65 μL of low melting point (LMP) agarose solution (final LMP agarose concentration 0.5%). Drops of each agarose-cell suspension were added on each pre-coated slide and placed for 1 h at 4 °C in the lysis solution (containing NaCl 2.225 M, Na_2_EDTA 88.9 mM, Tris 8.8 mM, NaOH 0.22 M, 10% DMSO, and 1% Triton X-100). Electrophoresis (1 V/cm) was performed for 20 min at 4 °C using an electrophoresis solution (Na_2_EDTA 1 mM and NaOH 300 mM, pH > 13). The solution on the slide was neutralized in triplicate (5 min every time) with Tris 0.4 M. Ethanol was used for fixation. Finally, the samples were stained with 2-(4-amidinophenyl)-1*H*-indole-6-carboxamidine (DAPI) (1 μg/mL) and analyzed using a fluorescence microscope (Eclipse Ni; Nikon Instruments Europe B.V., Badhoevedorp, The Netherlands). Over 150 nucleoids per sample (50 nucleoids per slide) stained with DAPI were scored using computerized image analysis (CaspLab, University of Wroclaw, Wroclaw, Poland). Cells containing damaged DNA appear like a comet with a bright head and a tail. In contrast, undamaged DNA appears as an intact nucleus with no tail. DNA damage was determined by comparing tail moments among groups.

### 4.7. Statistical Analysis

Descriptive statistical parameters (means and standard deviations of 8 samples for each experiment) were obtained for each studied variable. The means of independent variables were statistically compared among the groups. All analyses were performed using SPSS 26.0 (SPSS, Chicago, IL, USA). Differences were considered statistically significant at a probability level of <5%.

## 5. Conclusions

In conclusion, under the experimental conditions of this study, the exposure of HepG2 cells to 3 mg/L V administered as BMOV reduced cell viability, which was caused mainly by the effect of this metal in the damage of DNA on the cellular nucleus and mitochondria. These negative effects could be partially corrected by adding Cu (as CuCl_2_), which could act mainly by decreasing the damage to nuclear DNA. This positive association between these two metals could mediate the decrease in the toxicity of V and enhance its potential applications in therapy. Nevertheless, further studies are needed to better determine the effects arising from these interactions to establish the role of V as a micronutrient and to reduce its toxic effects.

## Figures and Tables

**Table 1:** The cellular metal content of HepG2 treated with metallic solutions for 32 h. Data are expressed as mean ± SD. Superscript letters denote significant differences: ^a^: vs. control cells; ^b^: vs. 3 mg V/L; ^c^: vs. 3 mg Cu/L.

	V (ng/10^6^ Cells)	Cu (ng/10^6^ Cells)
Control	4.8 ± 0.6	307 ± 110
3 mg/L V	50 ± 0.4 ^a^	503 ± 110 ^a^
3 mg/L Cu	4.84 ± 0.4 ^b^	5340 ± 1551 ^a,b^
3 mg/L Cu- 3 mg/L V	42 ± 0.2 ^a,c^	2255 ± 707 ^a,b,c^

**Table 2:** OTM for each group of HepG2 cells treated with the metallic solutions for 32 h. Superscript letters denote significant differences: ^a^: vs. control; ^b^: vs. 3 mg/L V. *p* < 0.05.

	Tail Moment Value
Control	0
3 mg/L V	0
3 mg/L Cu	35.6 ^a^
3 mg/L Cu–3 mg/L V	10.3 ^a,b^

## Data Availability

Not applicable.

## References

[1] Gummow B. (2011). Vanadium: Environmental Pollution and Health Effects. Encyclopedia of Environmental Health.

[2] Treviño S., Diaz A. (2020). Vanadium and Insulin: Partners in Metabolic Regulation. J. Inorg. Biochem..

[3] Orvig C., Caravan P., Gelmini L., Glover N., Herring F.G., Li H., McNeill J.H., Rettig S.J., Setyawati I.A. (1995). Reaction Chemistry of BMOV, Bis(Maltolato)Oxovanadium(IV), a Potent Insulin Mimetic Agent. J. Am. Chem. Soc..

[4] Sakurai H., Watanabe H., Tamura H., Yasui H., Matsushita R., Takada J. (1998). Insulin-Mimetic Vanadyl—Dithiocarbamate Complexes. Inorg. Chim. Acta.

[5] Thompson K.H., Lichter J., LeBel C., Scaife M.C., McNeill J.H., Orvig C. (2009). Vanadium Treatment of Type 2 Diabetes: A View to the Future. J. Inorg. Biochem..

[6] Goldfine A.B., Patti M.-E., Zuberi L., Goldstein B.J., LeBlanc R., Landaker E.J., Jiang Z.Y., Willsky G.R., Kahn C.R. (2000). Metabolic Effects of Vanadyl Sulfate in Humans with Non—Insulin-Dependent Diabetes Mellitus: In Vivo and in Vitro Studies. Metabolism.

[7] Li S.H., McNeill J.H. (2001). In Vivo Effects of Vanadium on GLUT4 Translocation in Cardiac Tissue of STZ-Diabetic Rats. Mol. Cell. Biochem..

[8] Ścibior A., Kurus J. (2019). Vanadium and Oxidative Stress Markers—In Vivo Model: A Review. Curr. Med. Chem..

[9] Aureliano M., De Sousa-Coelho A.L., Dolan C.C., Roess D.A., Crans D.C. (2023). Biological Consequences of Vanadium Effects on Formation of Reactive Oxygen Species and Lipid Peroxidation. Int. J. Mol. Sci..

[10] He X., Jarrell Z.R., Liang Y., Ryan Smith M., Orr M.L., Marts L., Go Y.-M., Jones D.P. (2022). Vanadium Pentoxide Induced Oxidative Stress and Cellular Senescence in Human Lung Fibroblasts. Redox Biol..

[11] Rivas-García L., Quiles J.L., Varela-López A., Arredondo M., Lopez P., Diéguez A.R., Montes-Bayon M., Aranda P., Llopis J., Sánchez-González C. (2020). In Vitro Study of the Protective Effect of Manganese against Vanadium-Mediated Nuclear and Mitochondrial DNA Damage. Food Chem. Toxicol..

[12] Ścibior A., Adamczyk A., Gołębiowska D., Niedźwiecka I., Fornal E. (2014). The Influence of Combined Magnesium and Vanadate Administration on the Level of Some Elements in Selected Rat Organs: V–Mg Interactions and the Role of Iron-Essential Protein (DMT-1) in the Mechanism Underlying Altered Tissues Iron Level. Metallomics.

[13] Chen L., Min J., Wang F. (2022). Copper Homeostasis and Cuproptosis in Health and Disease. Signal Transduct. Target. Ther..

[14] Ruiz L.M., Libedinsky A., Elorza A.A. (2021). Role of Copper on Mitochondrial Function and Metabolism. Front. Mol. Biosci..

[15] Plazas Guerrero C.G., Acosta Cota S.D.J., Castro Sánchez F.H., Vergara Jiménez M.D.J., Ríos Burgueño E.R., Sarmiento Sánchez J.I., Picos Corrales L.A., Osuna Martínez U. (2021). Evaluation of Sucrose-Enriched Diet Consumption in the Development of Risk Factors Associated to Type 2 Diabetes, Atherosclerosis and Non-Alcoholic Fatty Liver Disease in a Murine Model. Int. J. Environ. Health Res..

[16] Tsang T., Davis C.I., Brady D.C. (2021). Copper Biology. Curr. Biol..

[17] Rucker R.B., Cui C.T., Tchaparian E.H., Mitchell A.E., Clegg M., Uriu-Hare J.Y., Keen C.L., Roussel A.M., Anderson R.A., Favier A.E. (2002). Dietary Vanadium, P-ATPase-7A Expression and the Influence on Lysyl Oxidase and Cu Accumulation in Rat Skin and Liver. Trace Elements in Man and Animals 10.

[18] Ścibior A., Gołębiowska D., Adamczyk A., Kurus J., Staniszewska M., Sadok I. (2018). Evaluation of Lipid Peroxidation and Antioxidant Defense Mechanisms in the Bone of Rats in Conditions of Separate and Combined Administration of Vanadium (V) and Magnesium (Mg). Chem.-Biol. Interact..

[19] Treviño S., Díaz A., Sánchez-Lara E., Sanchez-Gaytan B.L., Perez-Aguilar J.M., González-Vergara E. (2019). Vanadium in Biological Action: Chemical, Pharmacological Aspects, and Metabolic Implications in Diabetes Mellitus. Biol. Trace Elem. Res..

[20] Arredondo M., Muñoz P., Mura C.V., Núñez M.T. (2003). DMT1, a Physiologically Relevant Apical Cu ^1+^ Transporter of Intestinal Cells. Am. J. Physiol.-Cell Physiol..

[21] Cordier W., Yousaf M., Nell M.J., Steenkamp V. (2021). Underlying Mechanisms of Cytotoxicity in HepG2 Hepatocarcinoma Cells Exposed to Arsenic, Cadmium and Mercury Individually and in Combination. Toxicol. Vitr..

[22] Wang P., Wu Q., Wang F., Zhang Y., Tong L., Jiang T., Gu C., Huang S., Wang H., Bu S. (2018). Evaluating Cellular Uptake of Gold Nanoparticles in HL-7702 and HepG2 Cells for Plasmonic Photothermal Therapy. Nanomedicine.

[23] Kammerer S., Küpper J.-H. (2018). Human Hepatocyte Systems for in Vitro Toxicology Analysis. J. Cell. Biotechnol..

[24] Sánchez-González C., Rivas-García L., López-Chaves C., Rodríguez-Nogales A., Algieri F., Gálvez J., Gómez-Aracena J., Vera-Ramírez L., Montes-Bayon M., Sanz-Medel A. (2014). Exposure to Bis(Maltolato)Oxovanadium(IV) Increases Levels of Hepcidin MRNA and Impairs the Homeostasis of Iron but Not That of Manganese. Food Chem. Toxicol..

[25] Sánchez-González C., Rivas-García L., Rodríguez-Nogales A., Algieri F., Gálvez J., Aranda P., Montes-Bayón M., Llopis J. (2021). Vanadium Decreases Hepcidin MRNA Gene Expression in STZ-Induced Diabetic Rats, Improving the Anemic State. Nutrients.

[26] Sanchez-Gonzalez C., Bermudez-Peña C., Guerrero-Romero F., Trenzado C.E., Montes-Bayon M., Sanz-Medel A., Llopis J. (2012). Effect of Bis(Maltolato)Oxovanadium (IV) (BMOV) on Selenium Nutritional Status in Diabetic Streptozotocin Rats. Br. J. Nutr..

[27] Sanchez-Gonzalez C., Bermudez-Peña C., Trenzado C.E., Goenaga-Infante H., Montes-Bayon M., Sanz-Medel A., Llopis J. (2012). Changes in the Antioxidant Defence and in Selenium Concentration in Tissues of Vanadium Exposed Rats. Metallomics.

[28] Samira M., Mounira T., Kamel K., Yacoubi M.T., Ben Rhouma K., Sakly M., Tebourbi O. (2018). Hepatotoxicity of Vanadyl Sulfate in Nondiabetic and Streptozotocin-Induced Diabetic Rats. Can. J. Physiol. Pharmacol..

[29] Sohrabi M., Gholami A., Azar M.H., Yaghoobi M., Shahi M.M., Shirmardi S., Nikkhah M., Kohi Z., Salehpour D., Khoonsari M.R. (2018). Trace Element and Heavy Metal Levels in Colorectal Cancer: Comparison Between Cancerous and Non-Cancerous Tissues. Biol. Trace Elem. Res..

[30] Sánchez-González C., Moreno L., Aranda P., Montes-Bayón M., Llopis J., Rivas-García L. (2022). Effect of Bis(Maltolato)Oxovanadium(IV) on Zinc, Copper, and Manganese Homeostasis and DMT1 MRNA Expression in Streptozotocin-Induced Hyperglycemic Rats. Biology.

[31] Bonnet C., Augustin S., Ellouze S., Bénit P., Bouaita A., Rustin P., Sahel J.-A., Corral-Debrinski M. (2008). The Optimized Allotopic Expression of ND1 or ND4 Genes Restores Respiratory Chain Complex I Activity in Fibroblasts Harboring Mutations in These Genes. Biochim. Biophys. Acta (BBA)-Mol. Cell Res..

[32] Danhelovska T., Kolarova H., Zeman J., Hansikova H., Vaneckova M., Lambert L., Kucerova-Vidrova V., Berankova K., Honzik T., Tesarova M. (2020). Multisystem Mitochondrial Diseases Due to Mutations in MtDNA-Encoded Subunits of Complex, I. BMC Pediatr..

[33] Bhatti J.S., Bhatti G.K., Reddy P.H. (2017). Mitochondrial Dysfunction and Oxidative Stress in Metabolic Disorders—A Step towards Mitochondria Based Therapeutic Strategies. Biochim. Biophys. Acta (BBA)-Mol. Basis Dis..

[34] Cobine P.A., Moore S.A., Leary S.C. (2021). Getting out What You Put in: Copper in Mitochondria and Its Impacts on Human Disease. Biochim. Biophys. Acta (BBA)-Mol. Cell Res..

[35] Peters K.G., Davis M.G., Howard B.W., Pokross M., Rastogi V., Diven C., Greis K.D., Eby-Wilkens E., Maier M., Evdokimov A. (2003). Mechanism of Insulin Sensitization by BMOV (Bis Maltolato Oxo Vanadium); Unliganded Vanadium (VO4) as the Active Component. J. Inorg. Biochem..

[36] Rivas-García L., Quiles J.L., Varela-López A., Giampieri F., Battino M., Bettmer J., Montes-Bayón M., Llopis J., Sánchez-González C. (2021). Ultra-Small Iron Nanoparticles Target Mitochondria Inducing Autophagy, Acting on Mitochondrial DNA and Reducing Respiration. Pharmaceutics.

